# Ion channel gating in cardiac ryanodine receptors from the arrhythmic RyR2-P2328S mouse

**DOI:** 10.1242/jcs.229039

**Published:** 2019-05-21

**Authors:** Samantha C. Salvage, Esther M. Gallant, Nicole A. Beard, Shiraz Ahmad, Haseeb Valli, James A. Fraser, Christopher L.-H. Huang, Angela F. Dulhunty

**Affiliations:** 1 Physiological Laboratory, University of Cambridge, Downing Street, Cambridge, CB2 3EG, UK; 2 Department of Biochemistry, University of Cambridge, Tennis Court Road, Cambridge, CB2 1QW, UK; 3 Eccles Institute of Neuroscience, John Curtin School of Medical Research, The Australian National University, 131 Garran Road, Acton ACT 2601, Australia; 4 Centre for Research in Therapeutic Solutions, Faculty of Science and Technology, University of Canberra, Bruce, ACT 2617, Australia

**Keywords:** RyR2 P2328S ion channel, P2328S-RyR2 mouse, Atrial fibrillation, Catecholaminergic polymorphic ventricular tachycardia, Cytoplasmic Ca^2+^ activation, Cytoplasmic Ca^2+^ inactivation, FKBP

## Abstract

Mutations in the cardiac ryanodine receptor Ca^2+^ release channel (RyR2) can cause deadly ventricular arrhythmias and atrial fibrillation (AF). The RyR2-P2328S mutation produces catecholaminergic polymorphic ventricular tachycardia (CPVT) and AF in hearts from homozygous RyR2^P2328S/P2328S^ (denoted RyR2^S/S^) mice. We have now examined P2328S RyR2 channels from RyR2^S/S^ hearts. The activity of wild-type (WT) and P2328S RyR2 channels was similar at a cytoplasmic [Ca^2+^] of 1 mM, but P2328S RyR2 was significantly more active than WT at a cytoplasmic [Ca^2+^] of 1 µM. This was associated with a >10-fold shift in the half maximal activation concentration (AC_50_) for Ca^2+^ activation, from ∼3.5 µM Ca^2+^ in WT RyR2 to ∼320 nM in P2328S channels and an unexpected >1000-fold shift in the half maximal inhibitory concentration (IC_50_) for inactivation from ∼50 mM in WT channels to ≤7 μM in P2328S channels, which is into systolic [Ca^2+^] levels. Unexpectedly, the shift in Ca^2+^ activation was not associated with changes in sub-conductance activity, S2806 or S2814 phosphorylation or the level of FKBP12 (also known as FKBP1A) bound to the channels. The changes in channel activity seen with the P2328S mutation correlate with altered Ca^2+^ homeostasis in myocytes from RyR2^S/S^ mice and the CPVT and AF phenotypes.

This article has an associated First Person interview with the first author of the paper.

## Abstract (precis)

**Summary:** The RyR2–P2328S mutation, precipitating potentially fatal arrhythmia in humans, can be attributed to leftward shifts in cytoplasmic Ca^2+^–dependent RyR2 activation and inactivation in the absence of adrenergic stimulation.

## INTRODUCTION

Mammalian heart rhythm depends on the activity of ion channels, ion transporters and Ca^2+^-binding proteins in the surface membrane, cytoplasm and intracellular sarcoplasmic reticulum (SR) Ca^2+^ store of cardiac myocytes. The intracellular Ca^2+^-handling proteins control Ca^2+^ release and contraction during systole, as well as diastolic Ca^2+^ re-uptake and storage in the SR. Mutations or acquired changes in these proteins lead to arrhythmia and heart failure (Bers, 2001). Mutations in the cardiac ryanodine receptor (RyR2), the ligand-gated SR Ca^2+^ release channel, can increase channel open probability during diastole, resulting in excess diastolic SR Ca^2+^ release, and lead to catecholaminergic polymorphic ventricular tachycardia (CPVT) (Laitinen et al., 2001; Lehnart et al., 2004; Priori et al., 2001; Swan et al., 1999), ventricular fibrillation (VF) (Cerrone et al., 2005; Jiang et al., 2007; Paech et al., 2014) or atrial fibrillation (AF) (Pizzale et al., 2008; Sumitomo et al., 2007). CPVT is an inherited channelopathy, and is characterised by often-fatal ventricular tachycardia exacerbated by physical or emotional adrenergic stress. To date, more than 150 different mutations have been found to disturb RyR2 ion channel function, and these account for 70–80% of CPVT cases. The mutations lead to abnormal SR Ca^2+^ handling and subsequent pro-arrhythmic SR Ca^2+^ ‘leak’ (Priori and Chen, 2011; Ronen and Lili, 2016; Sumitomo, 2016). In contrast to the typically monogenic nature of CPVT, VF and AF are multifactorial, but can also involve disrupted Ca^2+^ homeostasis. AF is the most common sustained arrhythmia, resulting in significant clinical morbidity and mortality (Benjamin et al., 1998; Davis et al., 2012; Kourliouros et al., 2009). Abnormally high diastolic cytoplasmic [Ca^2+^] triggers the surface membrane sodium calcium exchanger (NCX; herein referring to the isoform encoded by *SCL8A1*) to extrude Ca^2+^, generating a delayed after-depolarisation (DAD) as NCX imports three Na^+^ ions for every Ca^2+^ extruded. Triggered arrhythmogenic action potentials are generated when the DAD reaches the action potential threshold.

The molecular properties of RyR2 carrying CPVT and/or AF mutations that lead to diastolic Ca^2+^ leak are frequently studied using RyR2 channels expressed in HEK 293 cells (Jiang et al., 2005; Liu et al., 2013; Meli et al., 2011; Paavola et al., 2007). Mammalian models of RyR2 mutations are particularly useful because the ion channel is subjected to many post-translational modifications that also occur in patients, but only a few have been developed (Cerrone et al., 2005; Goddard et al., 2008; Lehnart et al., 2008; Shan et al., 2010; Shan et al., 2012). An advantage of animal models is that the ion channel properties can be directly correlated with changes in heart function and susceptibility to arrhythmia (Huang, 2017).

The RyR2-P2328S mutation is one of a few CPVT-related mutations that has been reported to also be associated with AF (Glukhov et al., 2015; Goddard et al., 2008; King et al., 2013; Laitinen et al., 2001; Lehnart et al., 2004; Salvage et al., 2015; Xiao et al., 2016; Zhang et al., 2011). We have generated a mouse model of the P2328S mutation; RyR2^P2328S/P2328S^ (RyR2^S/S^) (Goddard et al., 2008). The mutation lies within an RyR2 mutation ‘hot-spot’. This hot-spot, known as domain II or the central region, encompassing amino acids 2246–2534, is conserved across species and isoforms (Yano et al., 2005) and is included in the HD1 domain of RyR2 (Dhindwal et al., 2017). The RyR2^S/S^ mouse demonstrates the atrial and ventricular arrhythmic phenotype seen in patients (Goddard et al., 2008; Sabir et al., 2010; Zhang et al., 2011). The arrhythmia is associated with reduced action potential conduction velocity, reduced expression of Na_V_1.5 (also known as SCN5A) and reduced Na^+^ channel function (King et al., 2013; Ning et al., 2016; Salvage et al., 2015; Zhang et al., 2013). It is also associated with DAD phenomena (King et al., 2013), suggesting that RyR2 may be hyperactive. Indeed increased RyR2 sensitivity to cytosolic Ca^2+^ was indicated in a study of cellular Ca^2+^ handling (Goddard et al., 2008). P2328S RyR2 channels expressed in HEK 293 cells show increased activity at 150 nM cytoplasmic Ca^2+^, but only after protein kinase A (PKA) activation led to increased phosphorylation and reduced FKBP binding (Lehnart et al., 2004). Thus, in addition to its clinical phenotype, the P2328S mutation is of interest because of this reported loss of regulatory 12.6 kDa FK506-binding protein (FKBP12.6, also known as FKBP1B). In this context, the mutation is in the general proximity of a putative, albeit controversial, FKBP12- (also known as FKBP1A) and FKBP12.6-binding site (amino acids 2361–2496) within the central HD1 domain of RyR2 (Dhindwal et al., 2017; Laitinen et al., 2001; Lehnart et al., 2004; Marx et al., 2000; Xiao et al., 2016; Zissimopoulos and Lai, 2005). FKBP12 and FKBP12.6 are RyR2-associated proteins, reported to stabilise channel opening to the maximum conductance (Ahern et al., 1994; Brillantes et al., 1994; Lehnart et al., 2004; Marx et al., 2000; Wehrens et al., 2003). We find that FKBP12 and FKBP12.6 (hereafter FKBP12/12.6) removal from sheep RyR2 is correlated with increased sub-conductance activity in one RyR2 channelopathy linked to a mutation in the RyR2-associated regulatory protein CLIC-2 (Richardson et al., 2017). While the RyR2–FKBP12/12.6 association is widely documented, its role in modulating RyR2 channel activity is disputed (Meli et al., 2011; Shan et al., 2010; Xiao et al., 2007; Zissimopoulos and Lai, 2005).

Taking these considerations together, the ion channel properties of P2328S RyR2 from the RyR2^S/S^ mouse model have wide-reaching implications in cardiac dysfunction and physiology. Here, we examined the conductance and single-channel parameters of RyR2 from WT and RyR2^S/S^ mice, their cytoplasmic Ca^2+^ sensitivity, their FKBP12 association and S2814 and S2808 phosphorylation. [Note, that we refer to FKBP12 rather than FKBP12.6 as we found here that there was only one FKBP band associated with RyR2 in mouse heart, consistent with a previous report there is a 100-fold excess of FKBP12 compared to FKBP12.6 that is associated with RyR2 (Zissimopoulos et al., 2012).] Overall, we find that neither the full conductance of the channel, nor its sub-conductance properties are altered by the mutation. Ca^2+^ activation and, surprisingly, Ca^2+^ inactivation of mutant channels, are shifted to a lower cytoplasmic [Ca^2+^] so that the channel is maximally activated between 100 nM and 1 µM Ca^2+^. In contrast to channels expressed in HEK 293 cells, these changes are apparent without adrenergic stimulation and without loss of RyR2-associated FKBP12 or change in RyR2 S2814 or S2808 phosphorylation. The differences between the properties of channels expressed in mouse and in HEK 293 cells emphasise the importance of examining channels expressed in mature mammals.

## RESULTS

### Conductance of WT and P2328S RyR2 channels at 1 mM and 1 µM cytoplasmic [Ca^2+^]

**Fig. 1.**
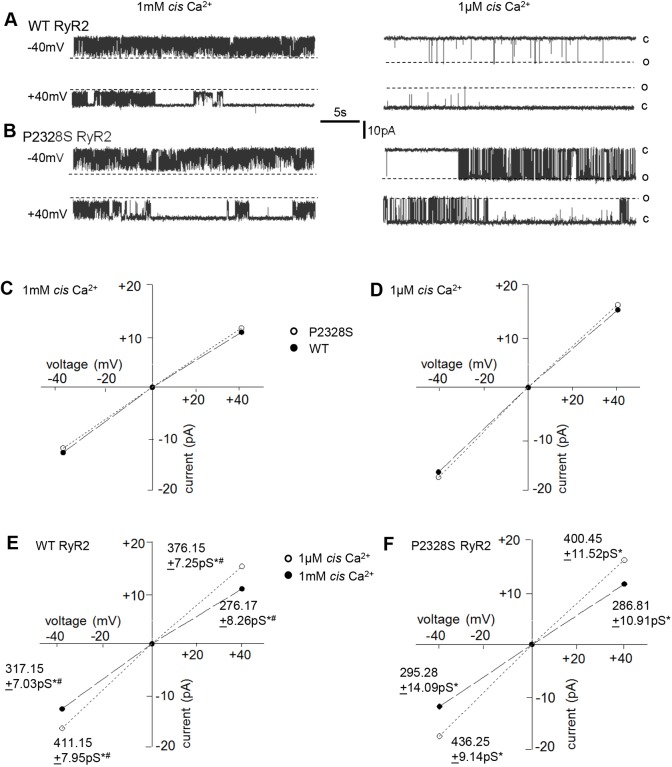
**The P2328S mutation enhances RyR2 channel activity when cytoplasmic [Ca^2+^] is 1 µM, without altering maximum channel conductance.** (A,B) Representative 25 s recordings from one WT RyR2 channel (A) and one P2328S RyR2 channel (B), with 1 mM Ca^2+^ (left panels) or 1 µM cytoplasmic Ca^2+^ (right panels), at −40 mV (upper record) or +40 mV (lower record). Broken lines (o) indicate maximum open currents, and solid lines (c) indicate the closed current levels. (C,D) Average maximum single channel currents plotted against voltage with 1 mM (C) or 1 μM (D) Ca^2+^. Dashed lines through filled circles connect WT data and dotted lines through open circles connect P2328S data. (E,F) Data from C and D replotted to emphasise the conductance difference between 1 mM with 1 μM Ca^2+^ in WT (E) or P2328S (F) RyR2. Dashed lines through filled circles connect 1 mM Ca^2+^ data, and dotted lines through open circles connect 1 µM Ca^2+^ data. Data points include mean±s.e.m. Error bars are within the symbol dimensions in all cases. Mean conductance (±s.e.m.) values are given beside their respective symbols. *n*=20 channels, WT, 1 mM Ca^2+^, −40 mV; *n*=24 channels, WT, 1 μM, −40 mV; *n*=22 channels, WT 1 mM Ca^2+^, +40 mV; *n*=23 channels, WT, 1 μM Ca^2+^, +40 mV. *n*=17 channels P2328S, 1 mM Ca^2+^, −40 mV; *n*=21 channels, P2328S, 1 μM Ca^2+^, −40 mV; *n*=18 channels, P2328S 1 mM Ca^2+^, +40 mV; *n*=22 channels, P2328S, 1 μM, +40 mV. ^#^*P*<0.05 between average data at −40 and +40 mV; *P<0.05 between conductances with 1 mM and 1 µM Ca^2+^. There was no significant difference between average WT and P2328S RyR2 conductances under any condition.

In initial experiments (experiment #1), WT and P2328S RyR2 activity was recorded continuously following channel incorporation into bilayers, starting with solutions on either side of the bilayer containing 1 mM Ca^2+^. The cytoplasmic [Ca^2+^] was lowered after 3 to 5 min to 1 μM (see Materials and Methods), a concentration that might exist in myocytes towards the end of diastole. There were no significant differences between the average maximum conductance of WT and P2328S channels recorded at the same bilayer voltage and cytoplasmic [Ca^2+^] (Fig. 1). However, currents in both channel types were greater with 1 μM cytoplasmic Ca^2+^ than with 1 mM Ca^2+^, likely due to removal of the partial pore block caused by Ca^2+^ binding (Hanna et al., 2014; Friel and Tsien, 1989; Gillespie et al., 2005). There was a small but significantly greater conductance in WT channels at −40 mV (with current flow from lumen to cytoplasm), which is also observed in sheep RyR2 (A.F.D., unpublished observations), and a similar trend in P2328S channels.

### The activity of WT and P2328S RyR2 channels at 1 mM and 1 µM cytoplasmic Ca^2+^ and ATP sensitivity

**Fig. 2.**
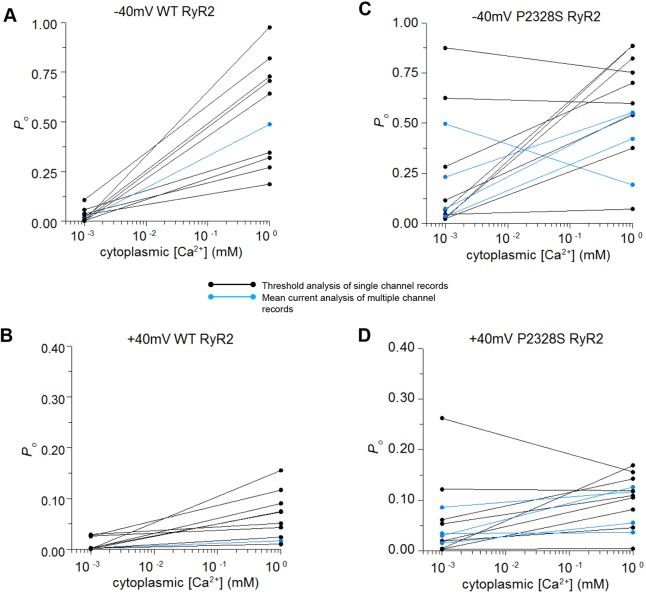
**The *P*_o_ of individual channels at 1 µM and 1 mM cytoplasmic [Ca^2+^] show substantial variability between P2328S channels with 1 µM Ca^2+^.** (A,B) WT channels. (C,D) P2328S channels. *P*_o_ at −40 mV plotted in A and C; *P*_o_ at +40 mV plotted in B and D. Black lines link individual channel data from bilayers containing one active channel (*P*_o_ determined with a threshold discriminator). Blue lines link measurements from bilayers with more than one channel opening (*P*_o_ estimated from *I*′*F*, the mean current normalised to maximum current, see Materials and Methods). Notably, *P*_o_ values derived from *I*′*F* fall within the range of values obtained from direct measurement of *P*_o_.

The biphasic cytoplasmic [Ca^2+^]–dependence of sheep and canine RyR2 channels shows a strong increase in activity between 1 μM and 10 μM Ca^2+^, a plateau between 10 µM and 1 mM and inactivation at concentrations ≫1 mM (Laver et al., 1995; Xu and Meissner, 1998). Thus, the decline in the activity of the mouse WT channel as shown in Fig. 1A when cytoplasmic Ca^2+^ was lowered from 1 mM to 1 μM was expected. The activity of the P2328S channel was similar to that of the WT channel with 1 mM cytoplasmic Ca^2+^, but was markedly higher when the [Ca^2+^] was lowered to 1 μM. This difference is reflected in the open probability (*P*_o_) of most individual channels (Fig. 2; Figs S1, S3 and S4: where lines connect data for individual channels at 1 mM and 1 µM Ca^2+^). Despite the usual (>10-fold) variability between the *P*_o_ of individual channels (Copello et al., 1997) at both 1 mM and 1 µM cytoplasmic Ca^2+^, the open probability of each WT channel was substantially lower with 1 µM than 1 mM Ca^2+^ (Fig 2A,B). The P2328S channels were more variable, with activity being lower at 1 µM than at 1 mM in 9 of 14 channels, changing very little in two channels and being higher for three channels (Fig 2C,D). As in Fig. 1 above, a robust voltage-dependence of *P*_o_ in these mouse RyR2 channels is notable in Fig. 2 and Figs S1, S3 and S4, with higher *P*_o_ at −40 than +40 mV in both WT and P2328S channels. The higher *P*_o_ at −40 mV suggests that the mutation would have the greatest impact when Ca^2+^ moves from the lumen of the SR into the cytoplasm, during systole and during diastolic Ca^2+^ leak. This voltage-dependence of *P*_o_ has been reported in sheep RyR2 (Dulhunty et al., 1999; Laver and Lamb, 1998; Sigalas et al., 2009), but is not apparent under all conditions (Dulhunty et al., 1999; Hewawasam et al., 2010; Laver and Lamb, 1998; Sigalas et al., 2009).

The *P*_o_ data in Fig. 2 is replotted on a logarithmic scale in Fig. S1 to show the spread of lower *P*_o_ values over an ∼100-fold range with 1 µM cytoplasmic Ca^2+^ at +40 mV. This in most cases exceeds the ∼10-fold range with 1 mM cytoplasmic Ca^2+^. The tighter range of values with 1 mM cytoplasmic Ca^2+^ may reflect more cohesive gating of *P*_o_ at maximally activated levels or a clustering due to the limiting *P*_o_ value of 1.00.

**Fig. 3.**
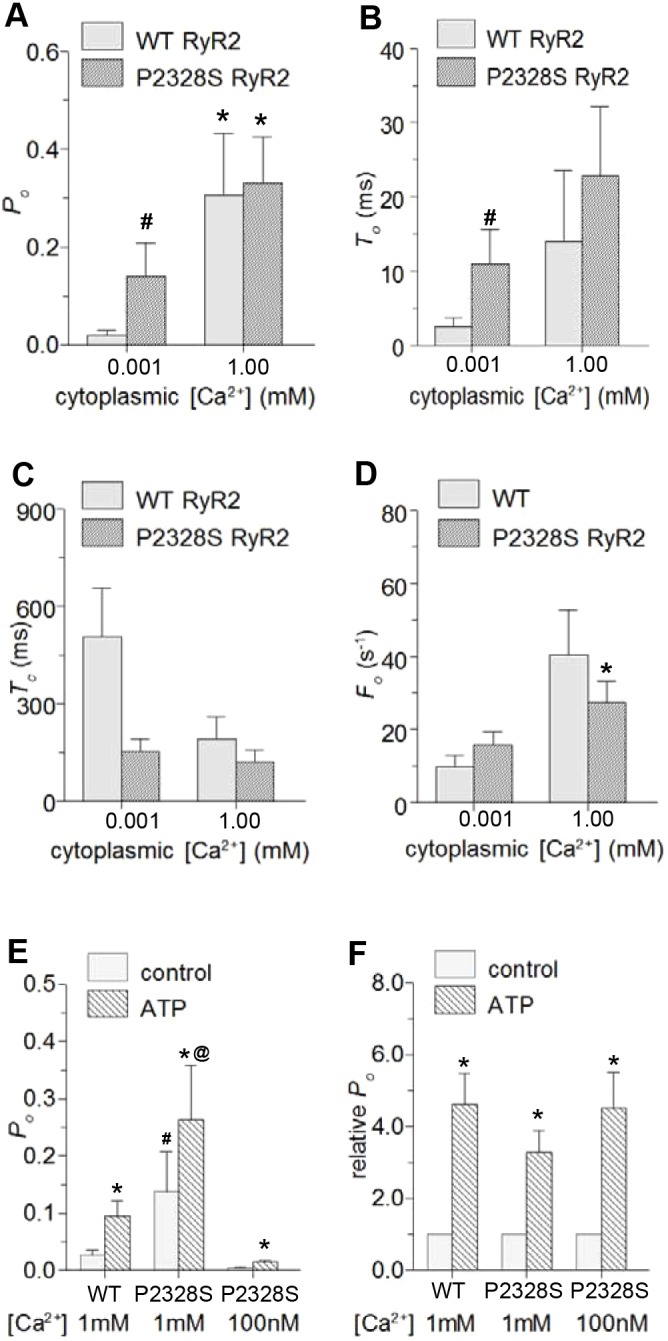
**Average gating parameters for WT and P2328S RyR2 channels exposed to 1 μM or 1 mM cytoplasmic Ca^2+^ and effects of ATP reveal significantly higher activity in P2328S with 1 μM Ca^2+^.** Average data (−40 and +40 mV pooled) for WT (light grey) and P2328S RyR2 (dark grey). (A–D) Mean±s.e.m. values for *P*_o_ (WT *n*=20; P2328S *n*=26), *T*_o_ (WT *n*=16; P2328S *n*=18), T_c_ (WT *n*=18; P2328S *n*=20) and *F*_o_ (WT *n*=18; P2328S *n*=20). *P<0.05 between 1 µM and 1 mM Ca^2+^. ^#^ *P*<0.05 between WT and P2328S. (E,F) Mean±s.e.m. data for WT (*n*=7) and P2328S (*n*=5) channels before (solid grey bars) and after (cross-hatched bars) exposure to 2 mM ATP, with cytoplasmic Ca^2+^ of 1 μM (*n*=7 WT; *n*=5 P2328S) or 100 nM (*n*=2 P2328S) as indicated. Absolute *P*_o_ (E); relative *P*_o_ (*F*). *P<0.05 for ATP being greater than control, ^#^*P*<0.05 for P2328S control with 1 μM Ca^2+^ being greater than WT or P2328S control at 100 nM Ca^2+^; ^@^*P*<0.05 for P2328S with 1 µM Ca^2+^ plus ATP being greater WT with 1 μM Ca^2+^ plus ATP or P2328S with 100 nM Ca^2+^ plus ATP.

Parameter values from single and multiple RyR2 channels are shown as the average *P*_o_ in Fig. 3A (assuming that *I′*_F_ for multiple channels is equal to the average *P*_o_ of the individual channels, see Materials and Methods). The mean open time (*T*_o_), mean closed time (*T*_c_) and event frequency (*F*_o_) could be measured only in single-channel recordings (Fig. 3B–D). There were no significant differences between WT and P2328S in any of the average parameters with 1 mM Ca^2+^. In marked contrast, at 1 μM Ca^2+^ there was a significant difference between WT and P2328S channels in average *P*_o_ *T*_o_ and *T*_c_, with P2328S channels having a higher *P*_o_, with longer openings and briefer closures. The higher WT *P*_o_ at 1 mM compared to 1 µM Ca^2+^ was due to a significantly longer *T*_o_, briefer *T*_c_ and higher *F*_o_. The average P2328S *P*_o_ was also higher with 1 mM than with 1 µM Ca^2+^ due to a significantly higher *F*_o_ and a trend towards longer open durations. The difference between *P*_o_ at 1 µM and 1 mM Ca^2+^ was substantially less in P2328S than in WT channels. Given the voltage-dependence of channel activity (Figs 1 and 2), the gating parameters are plotted separately for −40 and +40 mV in Fig. S2. The difference between the WT and P2328S *P*_o_ were significant at both potentials, but the other parameters mainly showed trends in the same directions as the significant changes in the combined data (Fig. 3A–D).

Overall the results in Fig. 3A–D and Fig. S2 show that increases in WT channel *P*_o_ between 1 µM and 1 mM Ca^2+^ are due to cytoplasmic Ca^2+^ increasing the duration and frequency of channel openings, while reducing the closed times. There were smaller differences between 1 µM and 1 mM Ca^2+^ in P2328S RyR2, with the significant increases in *P*_o_ and *F*_o_, and trends towards a longer *T*_o_ and shorter *T*_c_. The reduced sensitivity to this change in [Ca^2+^] in P2328S RyR2 could be due to either a reduced cytoplasmic Ca^2+^ sensitivity or a shift in the cytoplasmic Ca^2+^ dependence. These possibilities are explored in the following section.

Since the P2328 residue is contained within an RyR2 sequence that also binds ATP (Blayney et al., 2013), we examined the effect of the P2328S mutation on ATP activation. Cytoplasmic addition of 2 mM Na_2_ATP caused an increase in *P*_o_ in all WT and P2328S channels examined and a significant increase in the average *P*_o_ of WT RyR2 in the presence of 1 µM Ca^2+^ and of P2328S channels in the presence of 1 µM and 100 nM cytoplasmic Ca^2+^ (Fig. 3E). In the presence of ATP, the average *P*_o_ was significantly greater in P2328S with 1 μM Ca^2+^ than in WT channels or P2328S with 100 nM Ca^2+^. Prior to ATP addition, the *P*_o_ with 1 μM Ca^2+^ was significantly greater in P2328S than WT channels or P2328S channels with 100 nM Ca^2+^. However, relative increases in *P*_o_ were the same in all cases (Fig. 3F), indicating that ATP-activation per se is unaffected by the P2328S mutation.

Redox buffering was addressed in a subset of three WT and three P2328S ATP-activated channels. The GSH:GSSG ratio was set to an oxidising potential (see Materials and Methods) in cytoplasmic and luminal solutions to mimic cardiac oxidative stress (Oda et al., 2015). There was a trend towards the expected increase in *P*_o_ in WT channels (Pessah et al., 2002). The average WT *P*_o_ (data at +40 and −40 mV pooled) was 0.086±0.045 with ATP before, and 0.162±0.108 after, redox buffering. P2328S *P*_o_ was 0.191±0.078 before, and 0.163±0.051 after redox buffering (mean±s.e.m.). Although incomplete, these results suggest that the mutant channels may be less sensitive to oxidising cytoplasmic conditions than WT channels.

### Cytoplasmic Ca^2+^ sensitivity of WT and P2328S RyR2 channels

**Fig. 4.**
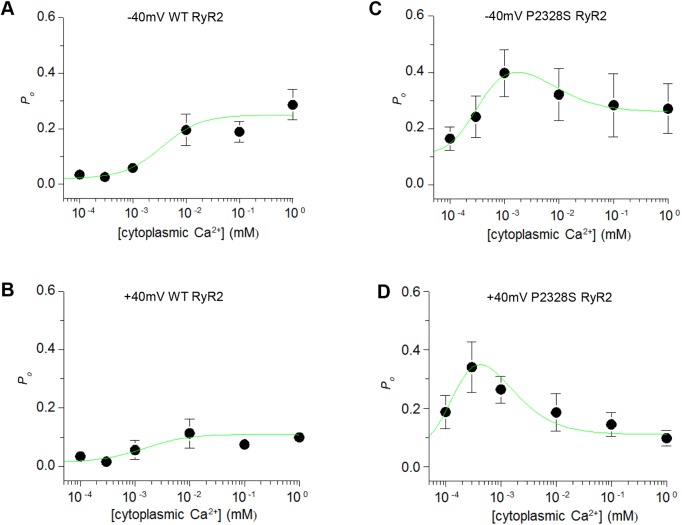
**The Ca^2+^-concentration dependence of *P*_o_ reveals substantial effects of the P2328S mutation on Ca^2+^-activation and Ca^2+^-inactivation.** (A,B) Average *P*_o_ from experiment #2 for WT RyR2 at −40 and +40 mV respectively. (C,D) Average *P*_o_ for P2328S RyR2 at −40 and +40 mV, respectively. Data points show mean±s.e.m. (s.e.m. markers are not visible when contained within the dimensions of the symbols). The numbers of observations were the same at −40 and +40 mV for each [Ca^2+^], but varied between each [Ca^2+^], depending on whether Ca^2+^ was initially reduced to 100 nM or 300 nM before subsequent concentration increases. For WT channels, *n*=x ([Ca^2+^]): 4 (100 nM); 3 (300 nM); 12 (1 µM); 8 (10 µM); 12 (100 μM); 8 (1 mM). For P2328S RyR2 channels, *n*=([Ca^2+^]): 10 (100 nM); 11 (300 nM); 10 (1 µM); 7 (10 µM); 12 (100 μM; 8 (1 mM). The green line Hill curves were fitted to the data using parameters in Table 1.

In experiment #2, the cytoplasmic incorporation solution containing 1 mM Ca^2+^ was replaced immediately after incorporation by perfusion with solutions containing 100 nM or 300 nM Ca^2+^ (see Materials and Methods). The [Ca^2+^] was then increased stepwise to 1 mM by addition of appropriate aliquots of CaCl_2_. The *P*_o_ for WT channels at 1 mM and 1 µM (Fig. 4) were not significantly different from those obtained in the first experiment. However, in contrast to experiment #1, the activity of most P2328S channels in experiment #2 declined at the higher Ca^2+^ concentrations (see individual channels in Figs S3 and S4). The average P2328S *P*_o_ was significantly less with 1 mM Ca^2+^ than with lower [Ca^2+^] at +40 mV (Fig. 4D), while at −40 mV there was a trend towards a lower *P*_o_ at 1 mM than 1 µM Ca^2^ (Fig. 4C).

**Table 1.**
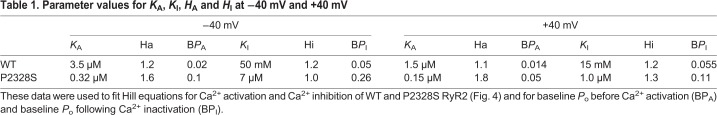
**Parameter values for *K*_A_, *K*_I_, *H*_A_ and *H*_I_ at −40 mV and +40 mV**

Although the difference between the two experiments may reflect the different order in which the solutions were changed, it is more likely that it reflects differences between individual P2328S channels. *P*_o_ was lower with 1 µM Ca^2+^ than 1 mM Ca^2+^ in all WT channels. By contrast, P2328S *P*_o_ in experiment #1 was greater or no different with 1 µM than 1 mM Ca^2+^ in three of 11 channels, while in experiment #2 *P*_o_ was greater or no different in seven of eight channels. Taking the two experiments together, the activity of ten of 19 P2328S channels was lower with 1 mM Ca^2+^ than with 1 µM Ca^2+^.

The average *P*_o_ values for WT channels described classical Ca^2+^ activation curves for RyR2 with *P*_o_ increasing steeply between 1 µM and 10 µM Ca^2+^ and reaching a plateau between 10 µM and 1 mM Ca^2+^ (Fig. 4A,B). Inactivation is not apparent in the WT data as it occurs when the cytoplasmic [Ca^2+^] is increased to non-physiological levels >1 mM (Laver et al., 1995). The Ca^2+^ dependence of *P*_o_ was substantially altered by the RyR2-P2328S mutation (Fig. 4C,D). The increase in P2328S RyR2 *P*_o_ with Ca^2+^ activation was shifted to a lower [Ca^2+^], with a maximum at 1 μM Ca^2+^ (−40 mV) or ∼300 nM (+40 mV). The average *P*_o_ declined with further increases in [Ca^2+^], likely reflecting Ca^2+^–dependent inactivation, and suggesting an unexpected shift in the inactivation curve into the physiological range of cytoplasmic [Ca^2+^].

Hill equations for Ca^2+^-dependent activation and inactivation (see Materials and Methods) were fitted to the data in Fig. 4. Affinity constants for activation and inactivation (*K*_A_ and *K*_I_, respectively) and Hill coefficients for activation and inhibition (*H*_A_ and *H*_I_, respectively) for the fitted curves are listed in Table 1. The P2328S mutation caused an ∼10-fold shift in Ca^2+^ activation. The affinity constant decreased from WT values of 3.5 μM to 0.32 μM Ca^2+^ in mutant channels at −40 mV, and from 1.5 μM in WT to 0.15 μM Ca^2+^ in mutant channels at +40 mV. Inactivation for P2328S RyR2 channels was shifted ∼1000-fold from millimolar range levels in WT channels to 7 µM at +40 mV or 1 μM at −40 mV. Additionally, the P2328S RyR2 inactivation curve at +40 mV (*K*_I_=1.0 μM) overlapped the activation curve (*K*_A_=0.15 μM). Therefore, the maximum *P*_o_ achieved of 0.35 was less than the maximum of 0.52 that would occur if activation had proceeded in the absence of inactivation. This is the first report of a RyR2 mutation causing such a dramatic shift in the Ca^2+^ dependence of inactivation. In contrast to the marked changes in *K*_I_ and *K*_A_, there was no change in the Hill coefficients, indicating that, as expected, the number of binding sites does not change.

The best fit of the Hill equations to the data required two assumptions. First, that there is a baseline *P*_o_ (B*P*_A_) when [Ca^2+^] is lower than the activating level, of 0.02 for WT RyR2 at −40 mV or 0.014 at +40 mV, or for P2328S of 0.1 at −40 mV or 0.05 at +40 mV. Second, that inactivation in P2328S RyR2 reduced *P*_o_ to a baseline level (B*P*_I_) of 0.26 at −40 mV or 0.11 at +40 mV, which is higher than for the WT channel B*P*_I_ of 0.05 at −40 mV and 0.055 at +40 mV.

### Sub-conductance activity in WT and P2328S RyR2 channels

**Fig. 5.**
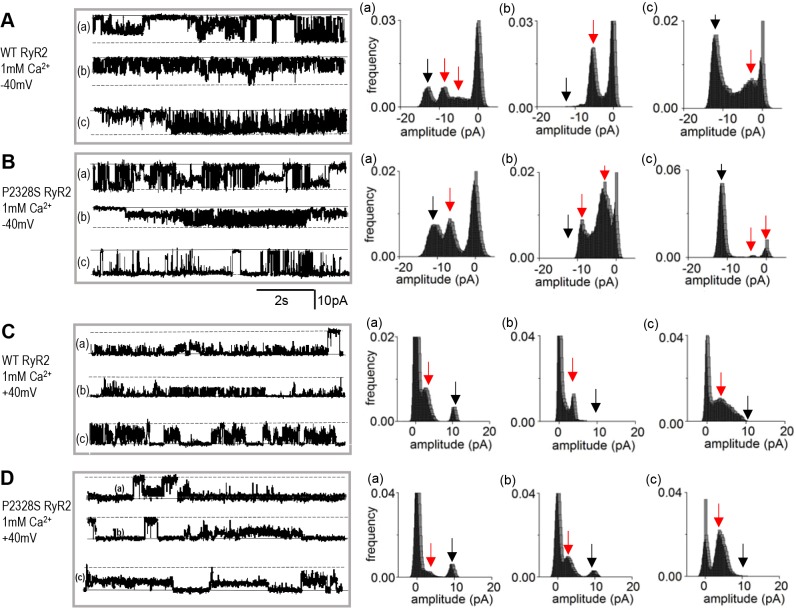
**Sub-conductance openings apparent in both WT and P2328S RyR2 channels with 1 mM cytoplasmic Ca^2+^.** Representative 8.5 s current recordings from three different bilayers (a, b and c) shown on the left, with corresponding amplitude histograms on the right. WT at −40 mV (A); P2328S at −40 mV (B); WT at +40 mV (C); P2328S at +40 mV (D). In current records, solid lines indicate the zero current and broken lines indicate maximum open current. In histograms, the black arrow points to the maximum single channel current and the red arrows indicate prominent sub-conductance levels.

**Fig. 6.**
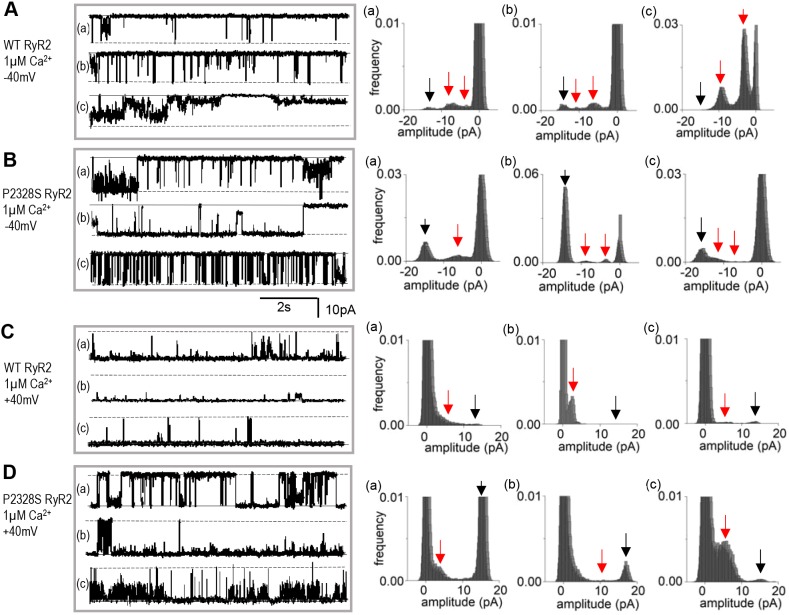
**Sub-conductance openings apparent in WT and P2328S RyR2 channel activity with 1 µM cytoplasmic Ca^2+^.** Representative 8.5 s current recordings from three different bilayers (a, b and c) shown on the left, with corresponding amplitude histograms on the right. WT at −40 mV (A); P2328S at −40 mV (B); WT at +40 mV (C); P2328S at +40 mV (D). In current records, solid lines indicate the zero current and the broken lines indicate maximum open current. In histograms, the black arrow points to the maximum single channel current and the red arrows indicate prominent sub-conductance levels.

Given the reported associations between arrhythmogenic mutations in RyR2, sub-conductance activity, phosphorylation and amounts of FKBP12 associated with RyR2 (see Introduction), we examined sub-conductance levels, FKBP12 binding to, and phosphorylation of, WT and P2328S RyR2 channels. Strong sub-conductance opening to current levels less than the maximum single-channel current was apparent in both channel types and is apparent in selected segments of activity from 24 different channels in Fig. 5 (with 1 mM cytoplasmic Ca^2+^) and Fig. 6 (with 1 µM cytoplasmic Ca^2+^). There were no consistent differences between the WT and P2328S RyR2 channels in sub-conductance levels or in amounts of sub-conductance activity. In each case, there are brief and very long openings to levels between 25% and 75% of the maximum current, with multiple levels, as well as one or two dominant levels. The same lack of difference is apparent in the longer recordings from 18 WT and 18 P2328S channels shown in Figs S3 and S4. Sub-conductance levels were generally scaled to the maximum current, so that the intervals between levels were least with 1 mM cytoplasmic Ca^2+^ and at +40 mV. Channel openings in WT and P2328S RyR2 are generally more clearly defined at −40 mV with 1 µM Ca^2+^ (Fig. 6) than with 1 mM cytoplasmic Ca^2+^ (Fig. 5). The lack of differences between the sub-conductance activity was not due to selection of the current segments in Figs 5 and 6 as they are clearly seen in the longer records of continuous activity from a larger number of channels, as shown in Figs S3 and S4. No quantitative evaluation of sub-conductance levels was attempted as we concluded that the activity was not substantially altered by the P2328S mutation.

### FKBP12 and FKBP12.6 association with WT and P2328S RyR2 channels

**Fig. 7.**
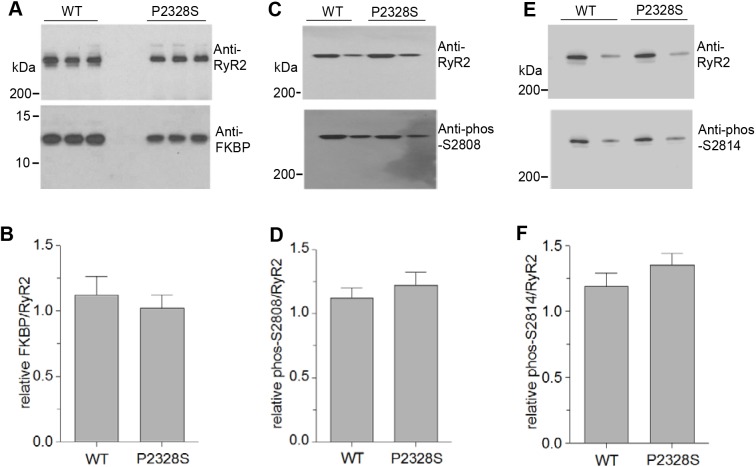
**Neither FKBP associated with RyR2 channels nor S2804 or S2814 phosphorylation are altered by the P2328S mutation.** (A) Representative blots of RyR2 and associated FKBP12 from WT and RyR2^S/S^ mouse hearts following co-IP of the FKBP–RyR2 complex with anti-RyR2 antibody, following by SDS-PAGE and immunoblotting (see Materials and Methods). (B) Average relative levels of FKBP12 bound to RyR2. FKBP12 band densities were normalised to the RyR2 band density for each lane, then expressed relative to the WT ratio. *n*=21 for WT and *n*=18 for P2328S data. (C–F) SR proteins separated via SDS-PAGE, subjected to western blotting and probed with antibodies to phosphorylated (phos) S2808 (C) or S2814 (E), then stripped and re-probed with anti-RyR2 as a loading control. Average data for RyR2 phosphorylation at S2808 (D) or S2814 (F). Band densities were normalised to total RyR2, then expressed relative to the WT phos-S2808/RyR2 or phos-S2814/RyR ratio. *n*=9 for S2808, *n*=6 for S2814. Data bars show mean±s.e.m.

FKBPs associated with the RyR2 was examined in the context of two controversial questions. First, regarding whether there is a correlation between sub-conductance activity and the amount of FKBP bound to RyR2 (Galfré et al., 2012; Lam et al., 1995) and, second, whether the binding of FKBPs to RyR2 is generally altered as a result of disease-associated mutations in RyR2 (Lehnart et al., 2004; Meli et al., 2011) or heart failure (Marx and Marks, 2002). Consistent with reported amounts of FKBP12.6 associated with RyR2 in mouse heart being ∼100-fold lower than FKBP12 (Zissimopoulos et al., 2012), we failed to see any convincing band corresponding to FKBP12.6 in western blots of WT or P2328S RyR2 mouse hearts. We routinely see bands corresponding to both isoforms in western blots of sheep and human heart (Richardson et al., 2017; Walweel et al., 2017). Blots of FKBP12 associated with RyR2 in SR vesicles suggest that there is no difference in its levels between WT or P2328S RyR2 (Fig. 7A) and there was no significant difference between the average relative amounts (Fig. 7B).

### Phosphorylation of WT and P2328S RyR2 channels

Hyperphosphorylation of RyR2 at S2808 and S2814 is associated with ventricular arrhythmia and FKBP dissociation from RyR2 (Dobrev and Wehrens, 2014). S2808 is hyperphosphorylated in humans and dogs with chronic AF (Vest et al., 2005). Human and rat S2804 and/or S2814 are basally phosphorylated and hyperphosporylated after β-adrenergic stimulation (Denniss et al., 2018; Li et al., 2013; Walweel et al., 2017). Phosphorylation of RyR2 S2804 and S2814 in SR vesicles from WT and RyR2^S/S^ mouse hearts was immuno–detected in western blots using antibodies that specifically recognise those residues only when they are phosphorylated. Both residues are basally phosphorylated in the absence of any experimental adrenergic treatment and there was no difference between the basal phosphorylation in WT or P2328S RyR2 in the individual blots (Fig. 7C,E) or in the average normalised data (Fig. 7D,F).

## DISCUSSION

Our findings demonstrate that mouse RyR2-P2328S channels are more active than WT RyR2 channels over the lower spectrum of physiologically relevant cytoplasmic Ca^2+^ concentrations (0.1–1 µM), with both Ca^2+^ activation and inactivation shifted, respectively, to nanomolar or micromolar Ca^2+^ levels. Notably this change in Ca^2+^ sensitivity occurred in the absence of adrenergic challenge. The channel properties indicate that P2328S RyR2 activity may be close to a balance between controlled and uncontrolled Ca^2+^ leak prior to adrenoreceptor activation and that such a challenge could tip the balance and trigger the cascade of events leading to potentiation of aberrant diastolic Ca^2+^ release. The greatly altered channel characteristics occurred without alterations in maximum conductance or sub-conductance activity and without change of FKBP12 binding or RyR2 S2808/S2814 hyperphosphorylation, again in the absence of adrenergic challenge. This suggests a mechanism for enhanced channel activity in P2328S-associated CPVT that does not depend on hyperphosphorylation or loss of FKBP12/12.6-mediated stabilisation of RyR2 channels. Notably our findings parallel cellular evidence showing that cardiomyocytes from RyR2^S/S^ hearts have higher incidences of spontaneous diastolic events than WT hearts without adrenergic challenge (Goddard et al., 2008; Zhang et al., 2011).

### Channel activity and its Ca^2+^ dependence in murine WT and P2328S RyR2 channels

The significantly higher *P*_o_ at 1 mM compared with 1 μM cytoplasmic Ca^2+^ in mouse WT RyR2 was similar to that for sheep and canine RyR2 (Sitsapesan and Williams, 1994; Xu and Meissner, 1998). The P2328S mutation reduced this difference between *P*_o_ at 1 µM and 1 mM Ca^2+^ (experiment #1) so that P2328S RyR2 channels were more active than WT RyR2 at 1 µM cytoplasmic Ca^2+^ (Figs 1–3). Activity in both channel types was significantly greater with current flow from lumen to cytosol at −40 mV.

The higher activity in P2328S RyR2 channels at 1 µM Ca^2+^ was due to a strong leftward shift to activation at lower [Ca^2+^], such that the channels were between 30% and 50% activated with 100 nM Ca^2+^ and fully activated with 1 µM Ca^2+^. This would lead to pronounced diastolic Ca^2+^ leak through P2328S channels that would be expected to be strongly pro-arrhythmic. A leftward shift in the Ca^2+^ activation curve is common with CPVT mutations (Meli et al., 2011; Xiao et al., 2016) and not surprising. However, the stronger ∼1000-fold shift in Ca^2+^ inactivation is a novel finding. This inactivation is within the normal range of systolic [Ca^2+^] and, hence, could truncate systolic Ca^2+^ release, thus reducing contraction. However, we predict that systolic Ca^2+^ release may not be affected, because the weaker shift in inactivation at −40 mV (current flow from lumen to cytoplasm) meant that *P*_o_ at −40 mV was similar in WT and P2328S channels at peak Ca^2+^ transient levels of ∼10–15 µM Ca^2+^. Consistent with this prediction, there are no reports suggesting reduced contraction with RyR2-P2328S mutations in humans or in the mouse model, while systolic Ca^2+^ transient amplitudes are similar in cardiomyocytes from WT and RyR2^S/S^ mice (Goddard et al., 2008; Zhang et al., 2011).

To fit Hill equations to the data, we assumed finite baseline activity levels. The baseline *P*_o_ at [Ca^2+^] below activation was higher in P2328S than WT channels at −40 mV and with Ca^2+^ inactivation, P2328S *P*_o_ decayed to a baseline level greater than the level before Ca^2+^ activation (Table 1), suggesting incomplete inactivation. The magnitude of the baseline *P*_o_ reported here would lead to massive diastolic Ca^2+^ leak. This would be less in myocytes where other factors including Mg^2+^ modulate channel activity. The effect of Mg^2+^ was not addressed in bilayer experiments because, in the absence of ATP and with cytoplasmic Ca^2+^ ≤1 μM, Mg^2+^ effectively reduces WT channel activity to such low values that quantification of channel activity is unreliable (Laver et al., 1997a; Xu et al., 1996). Nevertheless, we predict that even a smaller increase in baseline activity with the P2328S mutation would contribute significantly to increased diastolic Ca^2+^ leak.

### The impact of ATP activation and potential Mg^2+^ inhibition on P2328S channels

The ATP-binding site involves RyR1 residues M4954 and F4959 at the C-terminus of transmembrane S6 helix (des Georges et al., 2016). Equivalent RyR2 residues, M4884 and F4889, are presumably also adjacent to S6. P2328 is situated in the HD1 helical domain, which interacts extensively with regions near the ATP-binding site (Dhindwal et al., 2017). Indeed, Blayney et al. (2013) found high affinity ATP binding to the G2236–G2491 fragment (encompassing HD1, HD2 and P2328) (Blayney et al., 2013). That we did not see an effect of P2328S on ATP activation suggests that the P2328 residue is not involved in ATP binding. Similar ATP scaling of WT and P2328S activity suggests that the Ca^2+^ dependence of activation and inactivation might not be altered by ATP, although *P*_o_ would be higher.

The effects of the P2328S mutation on cytoplasmic Mg^2+^ and luminal Ca^2+^ and Mg^2+^ sensitivity, or other regulatory factors like H^+^, calmodulin, oxidation and nitrosylation were beyond the scope of this study. However it is interesting that Lehnart et al. (2004) found a right shift in cytoplasmic ‘Mg^2+^ inhibition’ of P2328S channels, which might appear inconsistent with the left shift we see in Ca^2+^ inactivation because cytoplasmic Mg^2+^ of ∼1 mM can occupy the Ca^2+^ inactivation site and inhibit RyR2. However, Mg^2+^ can bind to two independent cytoplasmic Ca^2+^-binding sites on RyR2, with effects that depend on cytoplasmic [Ca^2+^]. The low-affinity inhibitory (I1) site does not discriminate between Ca^2+^ and Mg^2+^, and Mg^2+^ binding to this site leads to Mg^2+^ inhibition with ≥100 μM cytoplasmic [Ca^2+^] (Laver, 2010; Walweel et al., 2014). This would indeed parallel the Ca^2+^ inactivation that we describe here. Mg^2+^ also binds to the higher affinity Ca^2+^ activation (A-) site, which selects Ca^2+^ over Mg^2+^ by ∼50–fold (Laver, 2010). 1 mM Mg^2+^ binds to the A-site and prevents Ca^2+^ in low micromolar range from binding and activating RyR2 (Laver, 2010), that is, it prevents Ca^2+^ activation rather than inhibiting the channel. The ‘Mg^2+^ inhibition’ described by Lehnart et al. (2004) using 150 nM cytoplasmic Ca^2+^ likely reflects Mg^2+^ binding to the A-site. A right shift in Mg^2+^ inhibition at the A-site in P2328S RyR2 is consistent with reduced Mg^2+^ binding as a result of enhanced Ca^2+^ affinity, allowing activation by 150 nM Ca^2+^. The relief of A-site Mg^2+^ binding would further enhance Ca^2+^ leak during diastole.

### Lack of alterations in FKBP association or phosphorylation in mouse P2328S RyR2 channels

The significant changes in the Ca^2+^ activation curve in mouse P2328S channels did not depend on adrenergic challenge, which is unlike what is seen for P2328S channels expressed in HEK 293 cells where increased activity with 150 nM Ca^2+^ was seen only after PKA phosphorylation (Lehnart et al., 2004). Our observations are more consistent with those of Zhang et al. (Zhang et al., 2011) who found significantly higher incidences of arrhythmia in isolated perfused RyR2^S/S^ hearts, compared to WT, prior to adding isoproterenol. In addition, diastolic Ca^2+^ release events are seen in isolated RyR2^S/S^, but not RyR2^+/S^ or WT atrial myocytes in the absence of isoproterenol (Goddard et al., 2008; Zhang et al., 2011). The difference between results with RyR2 P2328S channels expressed in HEK 293 cells and those with adult mice may not be surprising given that native channels from mouse are associated with regulatory proteins including triadin, junction and calsequestrin, which are lacking in the recombinant system. These contrasts underline the importance of examining the effects of mutations on RyR2 channels expressed in adult mammalian tissue.

The correlation between leaky RyR2 channels carrying CPVT mutations and FKBP binding to the channels is controversial. Reduced FKBP12.6 binding to recombinant RyR2 channels has been reported with P2328S, S2226L, R2474S and R4497C CPVT mutations (Lehnart et al., 2008; Wehrens et al., 2003), and increasing FKBP12.6 association with RyR2 can prevent aberrant SR Ca^2+^ release with AF and CPVT (Meli et al., 2011; Shan et al., 2012; Wehrens et al., 2003). Conversely, the CPVT R2474S RyR2 demonstrated increased FKBP12.6 affinity (Tiso et al., 2002), while enhanced FKBP12.6 binding did not alter arrhythmias in R4496C mice (Liu et al., 2006).

A complication with evaluating reported FKBP interactions with RyR2 was the assumption that RyR2 associates with FKBP12.6 alone, as FKBP12.6 was discovered in heart (Timerman et al., 1996). Therefore, many studies assumed that any FKBP bound to RyR2 was FKBP12.6, and only FKBP12.6 has been added to RyR2 in numerous functional studies. However many mammalian species (e.g. mouse, pig and rabbit), have more FKBP12 associated with RyR2 than FKBP12.6 (Zissimopoulos et al., 2012). A further complication is that FKBP12 and FKBP12.6 have different actions on RyR2; FKBP12 is a high-affinity sheep RyR2 activator, whereas FKBP12.6 has low efficacy, but antagonises the effects of FKBP12 (Galfré et al., 2012). There are no similar comparative reports for human or mouse RyR2. However we find substantial amounts of FKBP12 bound to healthy human (Walweel et al., 2017) and mouse RyR2 (Fig. 7). It may be more relevant to examine FKBP12 rather than, or combined with, FKBP12.6, association with RyR2 channels.

Altered sub-conductance opening has been attributed to altered FKBP12/12.6 binding to RyR2 (Lehnart et al., 2004; Marx et al., 2000; Richardson et al., 2017; Wehrens et al., 2003) or FKBP12 binding to RyR1 (Ahern et al., 1994; Brillantes et al., 1994). Our observations showing that neither the amount of FKBP12 bound to RyR2 nor sub-conductance activity are altered by the P2328S mutation again indicate a correlation between these parameters. That FKBP12 was not dissociated from P2328S RyR2 is consistent with a mutation in the HD1 domain (Dhindwal et al., 2017) which is distant from FKBP12 binding sites within RyR2 305–784 and 1815–1855 (Meli et al., 2011; Xiao et al., 2016) in both the linear sequence and 3D structure. Although FKBP12.6 stabilises HD2 (2982–3528), it does not alter HD1 (2110–2679) structure (Dhindwal et al., 2017).

As neither FKBP12 nor phosphorylation are important in the RyR2^S/S^ model, ‘intramolecular domain unzipping’ may lead to CPVT in RyR2 P2328S carriers. Interactions between N-terminal and central domains stabilise the channel closed state (Liu and Priori, 2007; Marks, 2002). As P2328S lies within the leucine-rich HD1 region (Dhindwal et al., 2017), the mutation could cause unzipping, hence decreasing the ability of the channel to remain closed and rendering it more sensitive to changes in cytosolic Ca^2+^ (Priori and Chen, 2011; Sumitomo, 2016)_,_ independently of FKBP12/12.6 or phosphorylation (Oda et al., 2015). Consistent with this, the closed times found here were significantly shorter in P2328S channels. Since our findings are in the absence of adrenergic activation, the changes could be more extreme during adrenergic stimulation, which increases channel activity at diastolic cytoplasmic [Ca^2+^] primarily through effects on luminal Ca^2+^ and Mg^2+^ regulation without altering A-site Ca^2+^ or Mg^2+^ regulation (Li et al., 2013). Provided the mutation did not alter the influence of phosphorylation on these luminal sites, we predict that adrenergic stimulation would further enhance channel activity at diastolic [Ca^2+^].

### The impact of changes in P2328S channel activity on Ca^2+^ efflux from the SR during diastole, DAD generation and arrhythmia

It is significant that, with diastolic cytoplasmic Ca^2+^ levels between 300 nM and 1 µM, *P*_o_ is three to five times greater in P2328S than WT channels. At 1 µM cytoplasmic Ca^2+^, the P2328S channel *P*_o_ is similar to the maximum Ca^2+^-activated *P*_o_ of WT channels with >10 µM cytoplasmic Ca^2+^. Therefore, robust local Ca^2+^ release from the RyR2^S/S^ mouse SR during diastole could lead to Ca^2+^ waves. Indeed, we previously reported ectopic Ca^2+^ transients in regularly stimulated RyR2^S/S^ murine hearts without adrenergic challenge, but not in their WT counterparts (Goddard et al., 2008). It is estimated that release of 30 to 40 µM of Ca^2+^ would be required to depolarise myocyte membranes to action potential threshold (Schlotthauer and Bers, 2000). Several factors may contribute to preventing this release from triggering an action potential in normal myocytes, providing a 3- to 4-fold safety margin (Schlotthauer and Bers, 2000). The three to five times greater activity of P2328S RyR2 channels during diastole could overcome this safety margin, allowing a greater local increase in cytoplasmic [Ca^2+^] and stronger depolarisation, triggering action potentials. It is likely that subclinical arrhythmic events, such as ectopic action potentials or non-sustained VT, occur without harming individuals and therefore remain undetected. Indeed, this may occur more frequently than anticipated as more-severe arrhythmic events, including polymorphic VT and sudden cardiac death, occur in CPVT patients at rest and during sleep (Allouis et al., 2005; D'Amati et al., 2005; Goddard et al., 2008; Postma et al., 2005). The incidence of severe arrhythmic events may be further increased by even relatively mild adrenergic challenge.

### General characteristics of single mouse WT and P2328S RyR2 channels

Single-channel analysis revealed some fundamental properties common to WT and P2328S RyR2 channels, which have not been reported, or which have been relatively overlooked in recent literature. First, channel conductance is significantly greater in WT channels when current flow is from lumen to cytoplasm (−40 mV), as during systole or diastolic ‘leak’, with a similar trend seen in P2328S channels. Second, a millimolar cytoplasmic [Ca^2+^] lowers channel conductance, due to Ca^2+^ blocking the pore (Hanna et al., 2014; Friel and Tsien, 1989; Gillespie et al., 2005). Third, *P*_o_ is highest when current flow is from the lumen to the cytoplasm. This has been attributed to ‘feed-through activation’ whereby luminal Ca^2+^ flows through the channel and binds to cytoplasmic Ca^2+^ activation sites (Sitsapesan and Williams, 1994; Tripathy and Meissner, 1996), or to Ca^2+^ binding to luminal Ca^2+^ activation sites (Laver, 2007). We see a greater *P*_o_ at −40 mV when both cytoplasmic and luminal solutions contain 1 mM Ca^2+^, so that both cytoplasmic and luminal Ca^2+^ activation sites would be occupied. Therefore, our results suggest that there is indeed a voltage, or direction of current flow sensor, within the transmembrane domain of the RyR2 that regulates channel gating and is not influenced by the P2328S mutation. The overall significance of this sensor would be to amplify channel activation through Ca^2+^-induced Ca^2+^ release during systole and during diastolic leak, particularly with the excess Ca^2+^ release imposed on the channel by the P2328S mutation.

Finally, our results demonstrate the well–documented (Copello et al., 1997) spread in parameter values in individual WT channels, which is exacerbated in P2328S channels as it is in human heart failure (Walweel et al., 2017). In experiment #1 (1 μM cytoplasmic Ca^2+^ at −40 mV), the range of WT *P*_o_ was 0.0016 to 0.11, compared to 0.0027 to 0.875 in P2328S channels. The WT variability is likely a consequence of the size of the RyR2 protein and number of regulatory sites, which may be vacated or occupied on each of the four subunits to different extents in each functional ion channel. Added to this, the increased P2328S variability was partly due to *P*_o_ remaining unchanged or increasing in many P2328S channels when cytoplasmic Ca^2+^ was reduced from 1 mM to 1 μM, in contrast to WT channels where *P*_o_ fell to lower values in all cases. A consequence of the variability is that, although changes in average gating parameters are often not significant, trends are indicative of underlying gating changes.

### Concluding comments

P2328S channels show increased sensitivity to cytosolic Ca^2+^ in the absence of adrenergic challenge. This could contribute significantly to the potentiation of aberrant SR Ca^2+^ leak under resting conditions, thus triggering cardiac arrhythmias and sudden cardiac death. The increase in open probability was observed in the absence of altered levels of FKBP12 binding or phosphorylation. This is in contrast to findings with FKBP12.6 and phosphorylation with the same mutation, and also some other mutations in RyR2 channels expressed in a HEK cell system, albeit following PKA stimulation (Wehrens et al., 2003; Lehnart et al., 2004; Meli et al., 2011). We uncovered a novel leftward shift in Ca^2+^-dependent inactivation towards the range of [Ca^2+^] achieved during systole, with incomplete inactivation. While this might not impact on the peak systolic Ca^2+^ transient, incomplete inactivation would contribute to maintaining cytoplasmic Ca^2+^ at a higher than normal level during diastole and add to the potential for DADs and arrhythmia. Overall, our results are consistent with the increased sensitivity to cytosolic Ca^2+^ suggested in RyR2^S/S^ mouse cells (Goddard et al., 2008; Zhang et al., 2011). It is worth considering that the location of P2328 and its proximity to Ca^2+^ activation and inactivation sites in the high-resolution structure of the RyR. The cytoplasmic Ca^2+^ and Mg^2+^ A-site and ATP-binding sites are located near the transmembrane channel pore region (des Georges et al., 2016; Jones et al., 2017) that interacts with HD1 residues containing P2328 (Dhindwal et al., 2017). A predicted Ca^2+^ and Mg^2+^ I1-binding site in residues 1873–1903 is located in the handle domain of RyR1 (Laver, 2018; Laver et al., 1997b), close in space to the HD domains (Yan et al., 2015). Therefore, structural changes in the HD1 domain caused by the P2328S mutation could sterically influence both the A and I1 Ca^2+^-binding sites producing the changes in channel activity reported here.

## MATERIALS AND METHODS

### Harvesting of mouse hearts

WT and homozygous *RyR2-P2328S* inbred 129/Sv mice, age matched across 3–7 months (in order to obtain a range that would represent a similar magnitude of age distribution in the human population), were killed by cervical dislocation in licensed institutional premises under the UK Animals (Scientific Procedures) Act 1986. Homozygous mice were used to ensure that all RyR2 channels were P2328S homotetramers and to reveal the full extent of the effect of the mutation. The hearts were rapidly excised and transferred to ice cold Krebs–Henseleit buffer (in mM: NaCl 119, NaHCO_3_ 25, KCl_4_, KH_2_PO_4_ 1.2, MgCl_2_ 1, CaCl_2_ 1.8, glucose 10 and Na-pyruvate 2; pH 7.4, 95% O_2_/5% CO_2_) to rinse and remove excess tissue and blood. The whole heart was then snap frozen in liquid N_2_. Hearts were couriered to Australia on dry ice and then stored at −80°C.

### Isolation of the RyR2 SR vesicle preparation

All steps of the SR vesicle preparation were performed on ice and/or at 4°C. For lipid bilayer experiments, five to seven hearts were homogenised in cardiac homogenising buffer (CHB, containing, in mM: sucrose 290, imidazole 10 and NaN_3_ 3, pH 6.9). The homogenate was then centrifuged at 12,000 ***g*** for 20 min, then the pellet discarded and the supernatant centrifuged at 43,000 ***g*** for 2 h. The pellet was resuspended in Buffer A (CHB plus 649 mM KCl) and centrifuged at 46,000 ***g*** for 1.5 h. This pellet was re-suspended in 125 µl per g of mouse heart (∼5 hearts) of buffer A plus protease inhibitor mixture and stored in 8 μl aliquots at −80°C for use in lipid bilayer experiments. All individual protease inhibitors were obtained from Sigma-Aldrich and were added to the final suspension at the following final concentrations: benzamidine hydrochloride hydrate (catalogue #B6506), 1.0 mM; pepstatin A (catalogue #P4265), 2.1 µM; leupeptin (catalogue #L2884), 1 µM; AEBSF/Pefabloc SC (catalogue #76307), 0.5 mM; calpain inhibitor I –(catalogue #A6185), 3 µM; calpain inhibitor II (catalogue no. A6060), 3 µM.

### Single-channel lipid bilayer recordings

Lipid bilayers were formed as previously described (Laver et al., 1995), by spreading a lipid mixture (phosphatidylethanolamine, phosphatidylserine and phosphatidylcholine in n-decane) across a 100 µm aperture in a partition separating the *cis* chamber from the *trans* chamber. SR vesicles were added to the *cis* solution so that, following incorporation, the cytoplasmic surface of SR and RyR2 faced that solution, which was then equivalent to the cytoplasmic solution. SR vesicles were incorporated using a cytoplasmic (*cis*) incorporation solution containing 230 mM caesium methanesulfonate (CsMS), 20 mM CsCl, 1 mM CaCl_2_ and 10 mM tetraethylsulfamide (TES) pH 7.4, and a luminal (*trans*) solution containing 30 mM CsMS, 20 mM CsCl, 1 mM CaCl_2_ and 10 mM TES pH 7.4. Following channel incorporation, CsMS was added to the *trans* side to equalise the concentration of the charge carrier [Cs^+^] in both solutions. Continuous current recording began at this point and continued for the duration of the experiment. A *cis* solution containing physiological cytoplasmic Ca^2+^ concentrations of 100 nM, 300 nM or 1 μM (with all other components identical to 1 mM Ca^2+^ *cis* solution) was introduced by a back-to-back 10 ml syringe aspiration-perfusion system designed to effectively replace the entire *cis* bathing solution. The Ca^2+^ concentration in the *cis* solution was later increased by adding appropriate amounts of CaCl_2_, which were again determined using a Ca^2+^ electrode. In addition, in many channels, after increasing [Ca^2+^] stepwise from 100 or 300 nM to 1 μM, 10 μM, 100 μM and 1 mM, we then re-perfused the cis chambers with the 100 or 300 nM Ca^2+^ solution and increased Ca^2+^ again to 1 μM or 10 μM. This allowed us to bracket at least some of the measurement and have more confidence in the results obtained for individual channels at lower [Ca^2+^]. All experiments were performed at a room temperature of 19±1^o^C.

Note that the *trans* (luminal) [Ca^2+^] was maintained at its physiological level of 1 mM throughout. Note also that the initial cytoplasmic [Ca^2+^] of 1 mM used for vesicle incorporation was higher than the cellular range of 100 nM to 10 µM, but was required to facilitate channel incorporation. Measurement of channel activity with 1 mM cytoplasmic Ca^2+^ was nevertheless of considerable interest because it provided an indication of whether the plateau of RyR2 Ca^2+^ activation was maintained up to that concentration or whether activity declined due to the lower affinity Ca^2+^ inactivation process.

In some channels the redox potential of the cytoplasmic and luminal solutions was clamped to an oxidising level of −180 mV by adding GSH:GSSG in the ratio of 0.95 mM:0.1 mM to each solution (Feng et al., 2000; Pessah et al., 2002), in the presence of cytoplasmic 1 μM Ca^2+^ and 2 mM ATP^2−^.

### Single-channel lipid bilayer electrophysiology and analysis

Electrodes in the solutions on either side of the bilayer were used to voltage clamp the bilayer potential to −40 or +40 mV (V*_cis_* – V*_trans_*) and to detect current flow through the channel. Bilayer potential was switched between −40 mV and +40 mV every 30 s. The open probability (*P*_o_), mean open time (*T*_o_) and mean closed time (*T*_c_) were measured, and sub-conductance activity analysed over 60 to 90 s of recordings in which only one channel opened in the bilayer, using the programs Channel 2 (now depreciated; developed by P. W. Gage and M. Smith, John Curtin School of Medical Research) or Channel 3 (developed by N. W. Laver, University of Newcastle, UK). Channel 3 software can be obtained from Professor D. R. Laver (University of Newcastle, Newcastle, NSW, Australia). Threshold levels for channel opening were set to exclude baseline noise at ∼20% of the maximum single-channel conductance. *P*_o_ alone was evaluated in recordings containing more than one channel from the mean current divided by the maximum open current to obtain a value for the fractional mean current (*I*′*F*), which reflects *P*_o_ and is equal to *P*_o_ under ideal conditions.

A curve describing the changes in *P*_o_ with Ca^2+^ concentration was constructed by multiplying Hill equations for activation and inhibition (Eqns 1 and 2, respectively) which were modified from Laver et al. (1995) to include non-zero *P*_o_ values as indicated before Ca^2+^ activation (B*P*_A_) and after Ca^2+^-dependent inhibition B*P*_I,_ as required to best fit the equations to the data.(1)
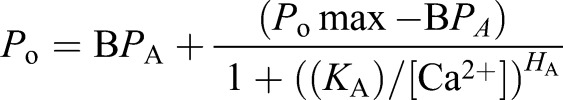
(2)
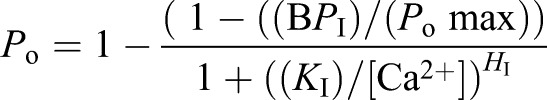
*P*_o max_ is the *P*_o_ of the achieved with maximal Ca^2+^ activation of the channel under the conditions of our experiment, *K*_A_ and *K*_I_ are the Ca^2+^ affinities of the activation and inhibition sites.

### Co-immunoprecipitation

Anti-RyR2 co-immunoprecipitation (Co-IP) of RyR2 complexes was performed to assess levels of FKBP12 and FKBP12.6 bound to RyR2 using the Pierce Co-IP kit and anti-RyR2 C3-33 antibody, following the manufacturer's instructions (Walweel et al., 2017). In brief, 100 μg of SR were diluted to 1 μg /μl in IP buffer (20 mM MOPS, 150 mM NaCl, 1 mM CaCl_2_, 1× cOmplete, EDTA-free protease inhibitor cocktail ; pH 7.4) with 5% glycerol and 0.1%Triton X-100 for 15 min on ice. Diluted SR samples were precleared with non-activated resin for 30 min at room temperature, with rotation. Precleared SR vesicles were separated from non-activated resin by centrifugation (1 min, 1000 ***g***), and incubated for 15 h at 4°C (with rotation) with anti-RyR2-antibody-bound resin in IP buffer. Unbound protein was removed by five washes of the complex with 200 μl of IP buffer, prior to elution of the bound RyR2 complexes from the anti-RyR2 antibody resin using 35 μl of 1×LDL sample buffer with reducing agent at 60°C for 10 min. Samples were then subject to SDS-PAGE and western blotting (below).

### SDS-PAGE and western blotting

SR vesicles (1–4 μg, for phosphorylated protein detection) or co-immunoprecipitates (for detection of FKBPs and RyR2), were subject to SDS-PAGE and western blotting as previously described (Laemmli, 1970; Towbin et al., 1979). Briefly, proteins were denatured in 1× Bolt LSL sample buffer with reducing agent (Life Technologies) at 60°C for 10 min. Samples and standards were loaded onto a 4–15% BOLT SDS-polyacrylamide gel (Thermo Fisher Scientific, Scorsby, Australia) and separated via electrophoresis using a Bolt Mini Gel system (Thermo Fisher Scientific) at 165 V until the dye from reached the bottom on the gel. The proteins were transferred onto PVDF membrane within a Bio-Rad Mini-Protean Tetra cell (Bio-Rad, Gladesville, Australia) in cold (4°C) transfer buffer (37 mM Tris, 140 mM glycine, 20% ethanol, no pH adjustment). To maximise transfer of large molecular mass proteins (such as RyR2), the transfer was carried out 1 h at 100 mV, and then for an additional 30 min at 200 mV. The PVDF membrane was blocked for 1–2 h at room temperature in blocking solution (3% BSA in PBS), and incubated with primary antibody (in PBS with 0.05% Tween 20 buffer) overnight at 4°C. Membranes were washed in PBS with 0.05% Tween 20 (5×), and incubated with appropriate secondary HRP-conjugated antibodies (IgG; in PBS with 0.05% Tween 20 for 1.5–2 h at room temperature). Blots were washed 5×in PBS+0.05% tween, once in PBS, prior to chemiluminescence detection.

Primary antibodies used were: anti-RyR2 C3-33 [MA3-916 ryanodine receptor monoclonal antibody (C3-33), used at a concertation of 1 μg/ml (Thermo Fisher Scientific)], anti-FKBP12 H5 [sc-133067 FKBP12 (H-5) mouse monoclonal antibody, used at a 1:200 dilution (Santa Cruz Biotechnology); this detects both 12.0 and 12.6 isoforms of FKBP] and RyR2 pSer28084 [A010-30AP RyR2 (pSer2808) rabbit polyclonal antibody, used at a 1:2000 dilution] and RyR2 pSer2814 [A010-31AP RyR2 (pSer2814) rabbit polyclonal antibody, used at a 1:5000 dilution], which detect the phosphorylated form of RyR2 residues S2808 or S2814, respectively (Badrilla, Leeds, UK). The specificity of the pSer2814 and pSer2814 antibodies for phosphorylated S2808 and S2814 (respectively) was validated by maximally phosphorylating (with PKA and CamKII) and dephosphorylating (with PP1) RyR2, as previously described (Walweel et al., 2017). Specificity of anti-RyR2 C3-33 for RyR2 was validated by probing native RyR2 (in SR vesicles) and purified mouse RyR2.

### Statistics

Average data is presented as mean±s.e.m. The significance of difference between various channel parameters for all channel data was assessed with one- or two-sided Student's *t*-tests as appropriate. The significance of differences in the co-IP and phosphorylation data were evaluated by performing one-way ANOVA with Tukey post hoc testing. Differences were considered significant with *P*<0.05.

## Supplementary Material

Supplementary information
