# Supplementary material for: Ion channel gating in cardiac ryanodine receptors from the arrhythmic RyR2-P2328S mouse
Source: J Cell Sci. 2019 May 21;132(10):jcs229039. doi: 10.1242/jcs.229039 (PMC6550012; doi:10.1242/jcs.229039)
Supplement: Supplementary information [file joces-132-229039-s1.pdf]

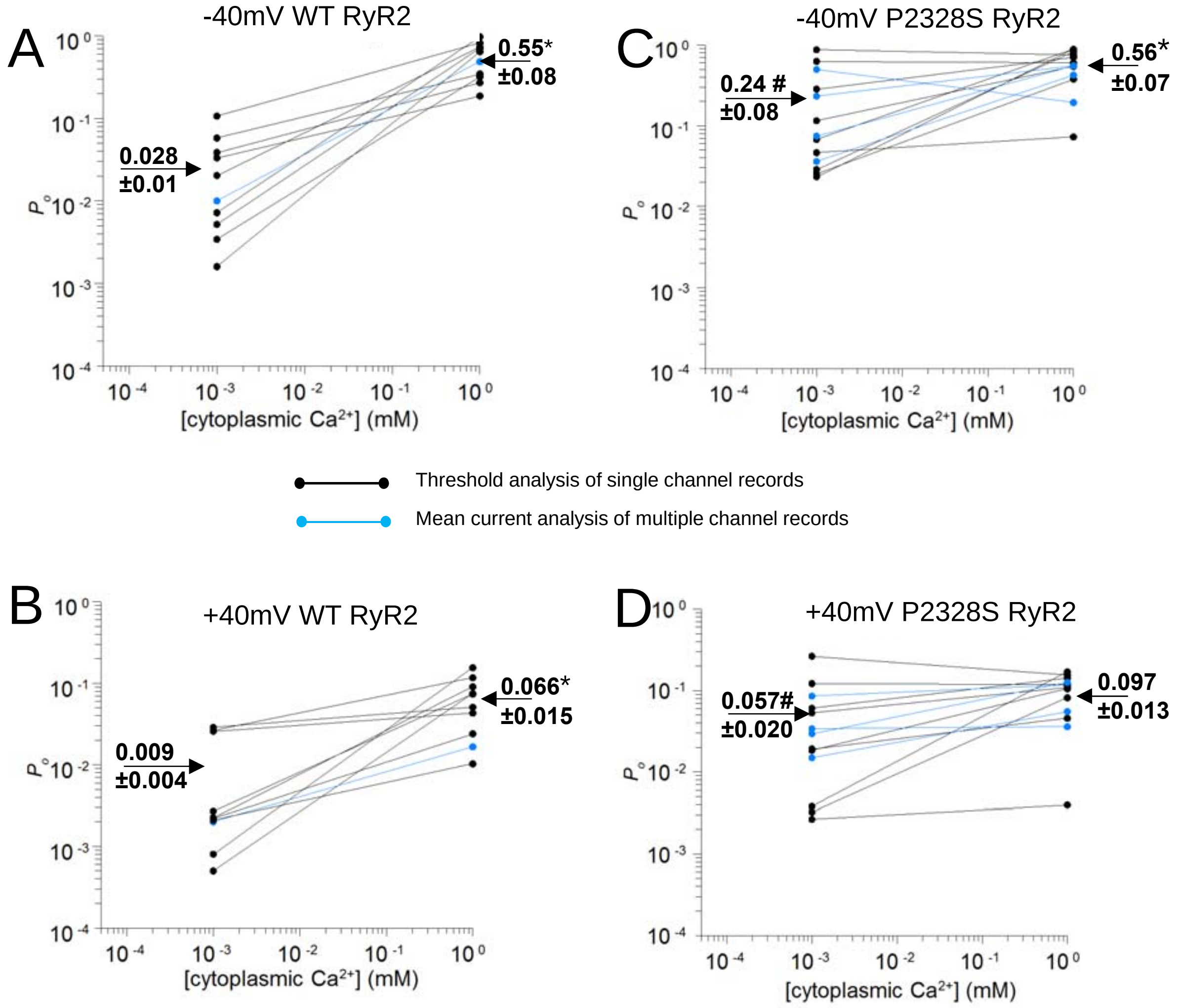

Figure S1. **P_o of individual channels at 1 µM and 1 mM cytoplasmic [Ca^{2+}] plotted on a logarithmic scale to reveal the spread of data at 1 µM cytoplasmic [Ca^{2+}]. (A)** and **(B)** show data for WT channels. **(C)** and **(D)** show data for P2328S RyR2 channels. P_o values for recordings at -40 mV are shown in **(A)** and **(C)**, while P_o values for recordings at +40 mV are shown in **(B)** and **(D)**. The black lines link P_o measurements from bilayers having one active channel, where P_o was determined using the threshold discriminator method. The blue lines link measurements obtained from bilayers with more than one channel opening at the same time, where *Po* is derived from I'_F (mean current normalized to maximum current, as described in the Methods). In **(A)** – **(D)**, average values for P_o at 1 µM cytoplasmic Ca^{2+} are shown to the left of the data points and average values for P_o at 1 mM cytoplasmic Ca^{2+} are shown to the right of the data points. The asterisk (*) indicates a significant difference between average P_o at 1 µM Ca^{2+} and at 1 mM Ca^{2+}. The # symbol indicates a significant difference between average P_o of WT and P2328S RyR2 channels.

Journal of Cell Science • Supplementary information

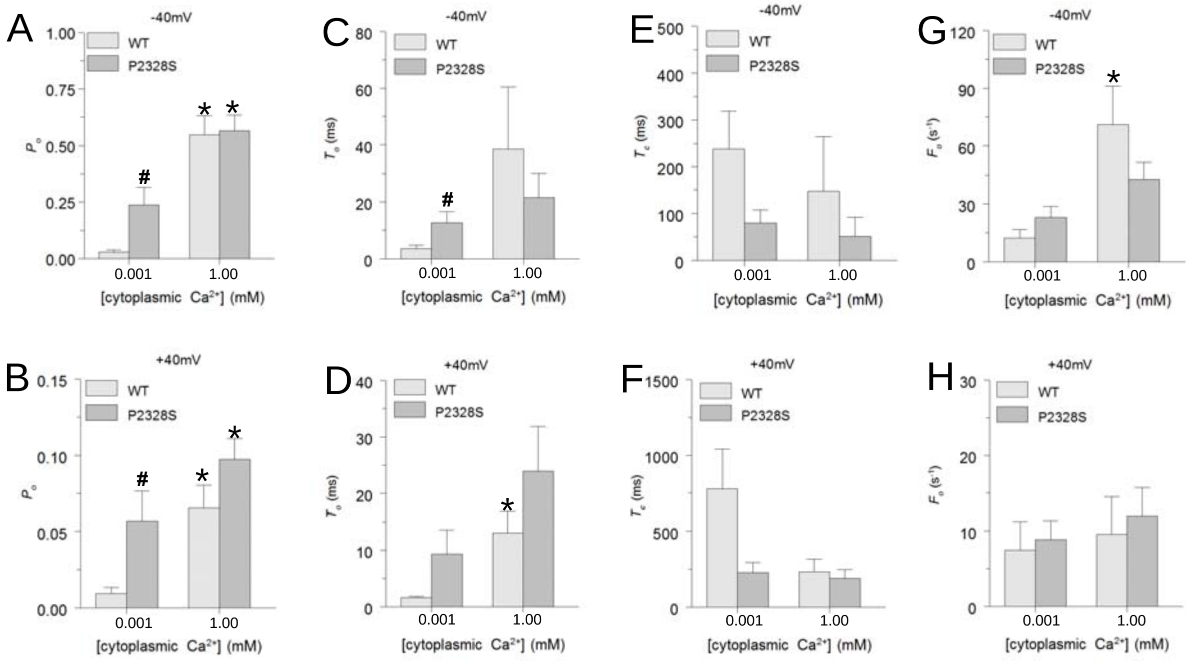

Figure S2. Average gating parameters of WT and P2328S RyR2 channels exposed to 1 μM and 1 mM cytoplasmic Ca^{2+} at -40 mV and at +40 mV. Average data is compared for WT (light grey bars) and P2328S RyR2 (dark grey bars).
(**A**), (**C**), (**E**) and (**G**) show average values at -40 mV for P_o (WT n = 10; P2328S n=13), T_o (WT n = 8; P2328S n=9), T_c (WT n = 9; P2328S n=10) and F_o (WT n = 9; P2328S n=10), respectively. (**B**), (**D**), (**F**) and (**H**) show average values, at +40 for P_o (WT n = 10; P2328S n=13), T_o (WT n = 9; P2328S n=10), T_c (WT n = 9; P2328S n=10) and F_o (WT n = 9; P2328S n=10), respectively. Data is presented as mean ± SEM. The asterisk (*) indicates a significant difference between average data at 1 μM Ca^{2+} and at 1 mM Ca^{2+}. The # symbol indicates a significant difference between average data for WT and P2328S channels

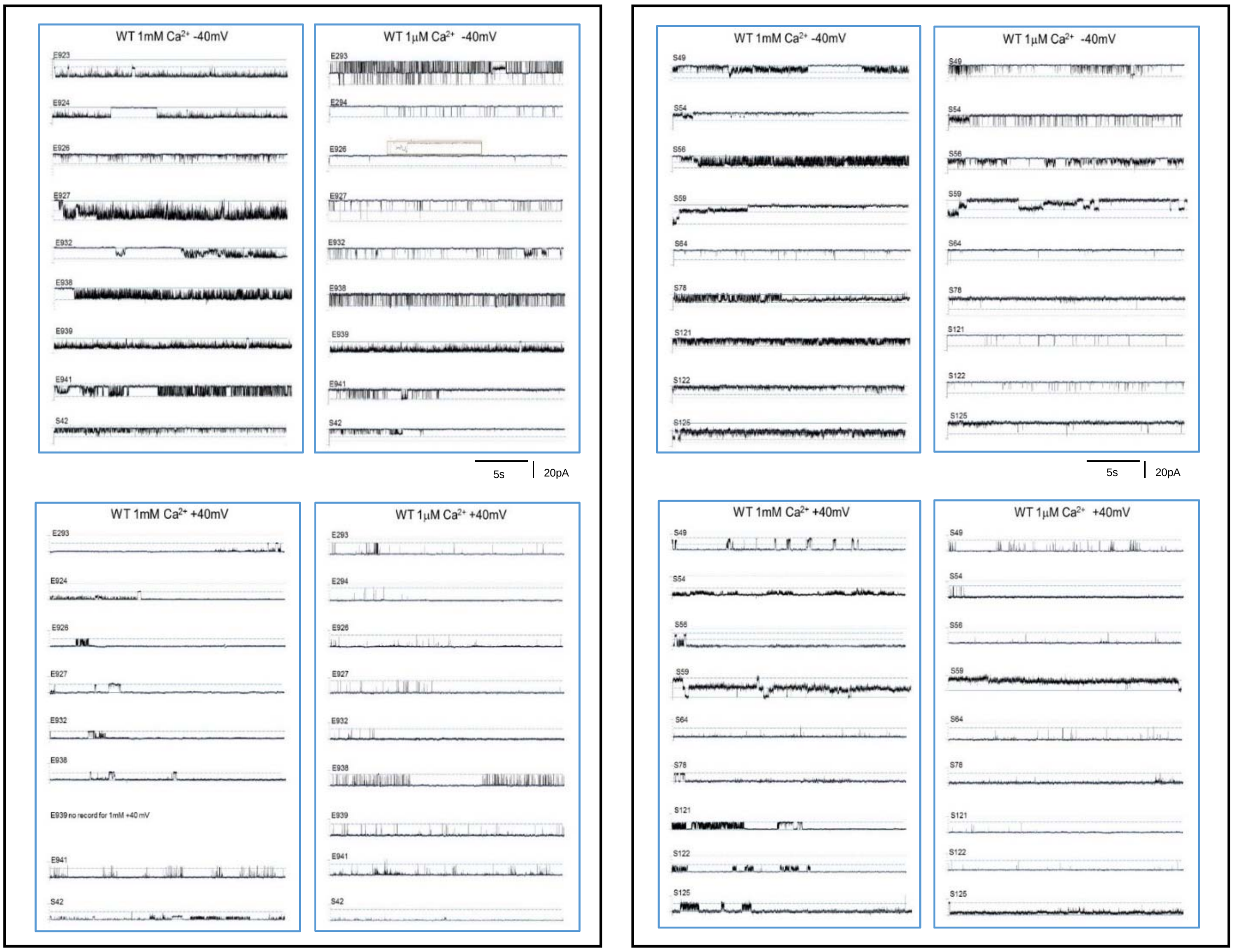

Figure S3. Examples of currents recorded from 18 WT channels used for analyses of channel activity with 1 mM and 1 µM cytoplasmic Ca^{2+} presented in manuscript Figures 1 to 6. In each panel, records from the same channel are aligned horizontally to allow direct comparisons of the channel activity under each condition. Currents shown on the left hand side of each panel were recorded with 1 mM cytoplasmic Ca^{2+}, while those on the right hand side were recorded with 1 µM cytoplasmic Ca^{2+}. Currents shown on the top half of each panel were recorded at -40 mV, while those in the lower half were recorded at +40 mV.

Journal of Cell Science • Supplementary information

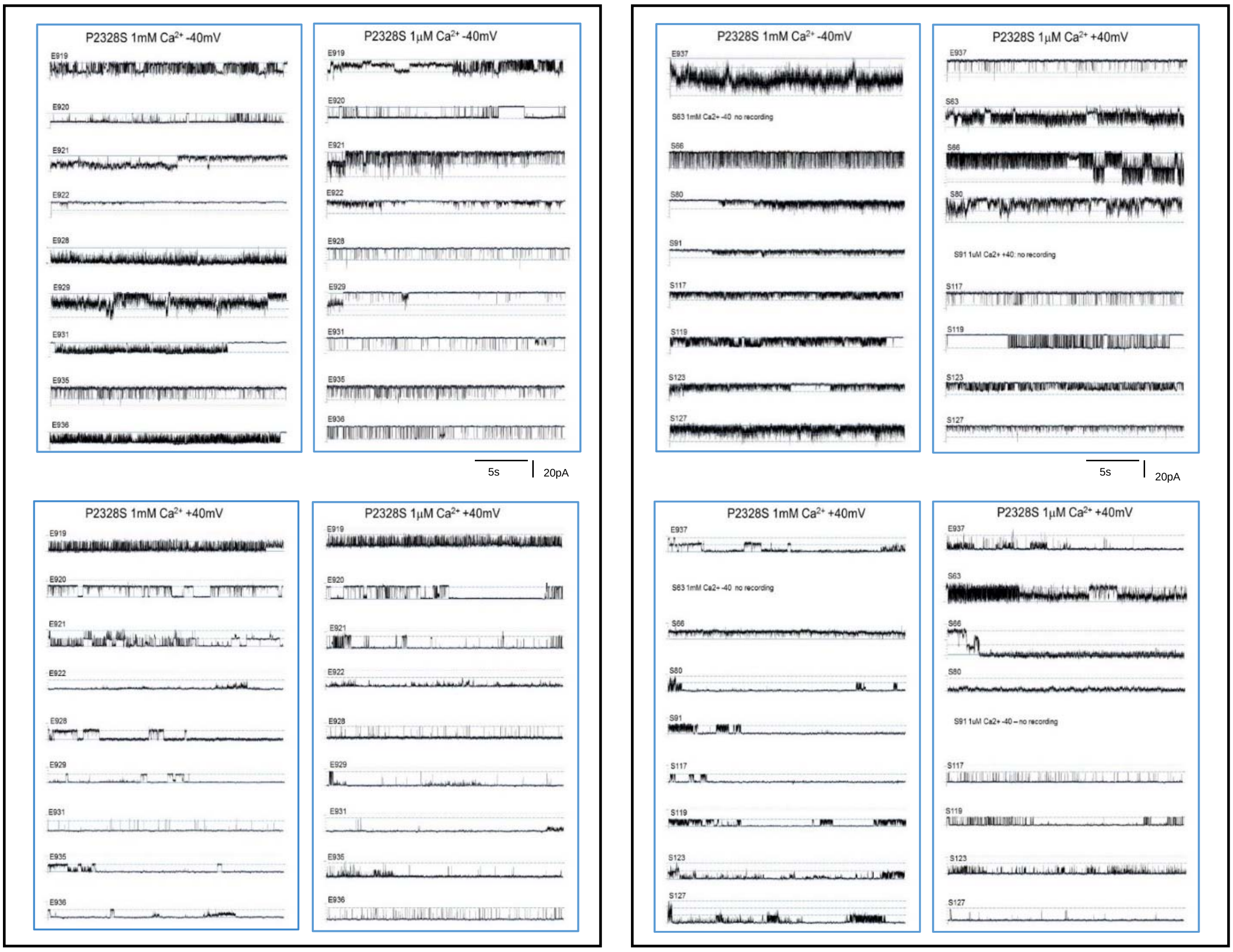

Figure S4. Examples of current recordings from 18 P2328S RyR2 channels used for analyses of channel activity with 1 mM and 1 µM cytoplasmic Ca^{2+} presented in manuscript Figures 1 to 6. In each panel, records from the same channel are aligned horizontally to allow direct comparisons of the channel activity under each condition. Currents shown on the left hand side of each panel were recorded with 1 mM cytoplasmic Ca^{2+}, while those on the right hand side were recorded with 1 µM cytoplasmic Ca^{2+}. Currents shown on the top half of each panel were recorded at -40 mV, while those in the lower half were recorded at +40 mV.

Journal of Cell Science • Supplementary information
